# Integrating Cellular and Bioprocess Engineering in the Non-Conventional Yeast *Yarrowia lipolytica* for Biodiesel Production: A Review

**DOI:** 10.3389/fbioe.2017.00065

**Published:** 2017-10-17

**Authors:** Dongming Xie

**Affiliations:** ^1^Massachusetts Biomanufacturing Center, Department of Chemical Engineering, University of Massachusetts Lowell, Lowell, MA, United States

**Keywords:** *Yarrowia lipolytica*, fatty acids, biodiesel, metabolic engineering, bioprocess engineering

## Abstract

As one of the major biofuels to replace fossil fuel, biodiesel has now attracted more and more attention due to its advantages in higher energy density and overall less greenhouse gas generation. Biodiesel (fatty acid alkyl esters) is produced by chemically or enzymatically catalyzed transesterification of lipids from microbial cells, microalgae, oil crops, or animal fats. Currently, plant oils or waste cooking oils/fats remain the major source for biodiesel production *via* enzymatic route, but the production capacity is limited either by the uncertain supplement of plant oils or by the low or inconsistent quality of waste oils/fats. In the past decades, significant progresses have been made on synthesis of microalgae oils directly from CO_2_ *via* a photosynthesis process, but the production cost from any current technologies is still too high to be commercialized due to microalgae’s slow growth rate on CO_2_, inefficiency in photo-bioreactors, lack of efficient contamination control methods, and high cost in downstream recovery. At the same time, many oleaginous microorganisms have been studied to produce lipids *via* the fatty acid synthesis pathway under aerobic fermentation conditions, among them one of the most studied is the non-conventional yeast, *Yarrowia lipolytica*, which is able to produce fatty acids at very high titer, rate, and yield from various economical substrates. This review summarizes the recent research progresses in both cellular and bioprocess engineering in *Y. lipolytica* to produce lipids at a low cost that may lead to commercial-scale biodiesel production. Specific technologies include the strain engineering for using various substrates, metabolic engineering in high-yield lipid synthesis, cell morphology study for efficient substrate uptake and product formation, free fatty acid formation and secretion for improved downstream recovery, and fermentation engineering for higher productivities and less operating cost. To further improve the economics of the microbial oil-based biodiesel, production of lipid-related or -derived high-value products are also discussed.

## Introduction

As the world population continuously increases, we are facing a challenge of overusing petroleum as energy sources and the enhanced environmental requirements to prevent global warming. If fossil fuels are replaced by biofuels, the total CO_2_ emissions could be reduced by 60–90% (Hasunuma et al., 2012). The two most common examples of biofuels are bioethanol and biodiesel (Kricka et al., 2014). So far, ethanol is still the most significant sources of biofuels that are mainly produced from corn starch or sugarcanes in USA, Brazil, and many other countries. However, the goal of advanced biofuel production should be using lignocellulosic biomass to produce biofuels with a compatibility with the current fossil fuels and the same quality.

As a promising source of making biofuels, lignocellulosic biomass is enriched with a structural material that comprises cellulose, hemicellulose, and lignin. Currently, the major sources of lignocellulosic biomass for biofuel production are from corn stover, switchgrass, wood chips, etc. Comparing to the well-studied biofuel production from corn starch and sugar canes, making biofuels from lignocellulosic biomass has the advantages in requiring significantly lower raw material cost and providing more substrate diversity. However, lignocellulosic biofuels may also require a much more complicated pretreatment to obtain C5/C6 sugars that can be directly used by microorganisms to convert into biofuels (Ledesma-Amaro et al., 2015a). For this purpose, metabolic pathways for various types of fuels, including fermentative and non-fermentative alcohols, fatty acids-derived fuels, and isoprenoid-derived fuels, should be reconstructed in a selected strain (Kang and Lee, 2015).

**Figure 1:**
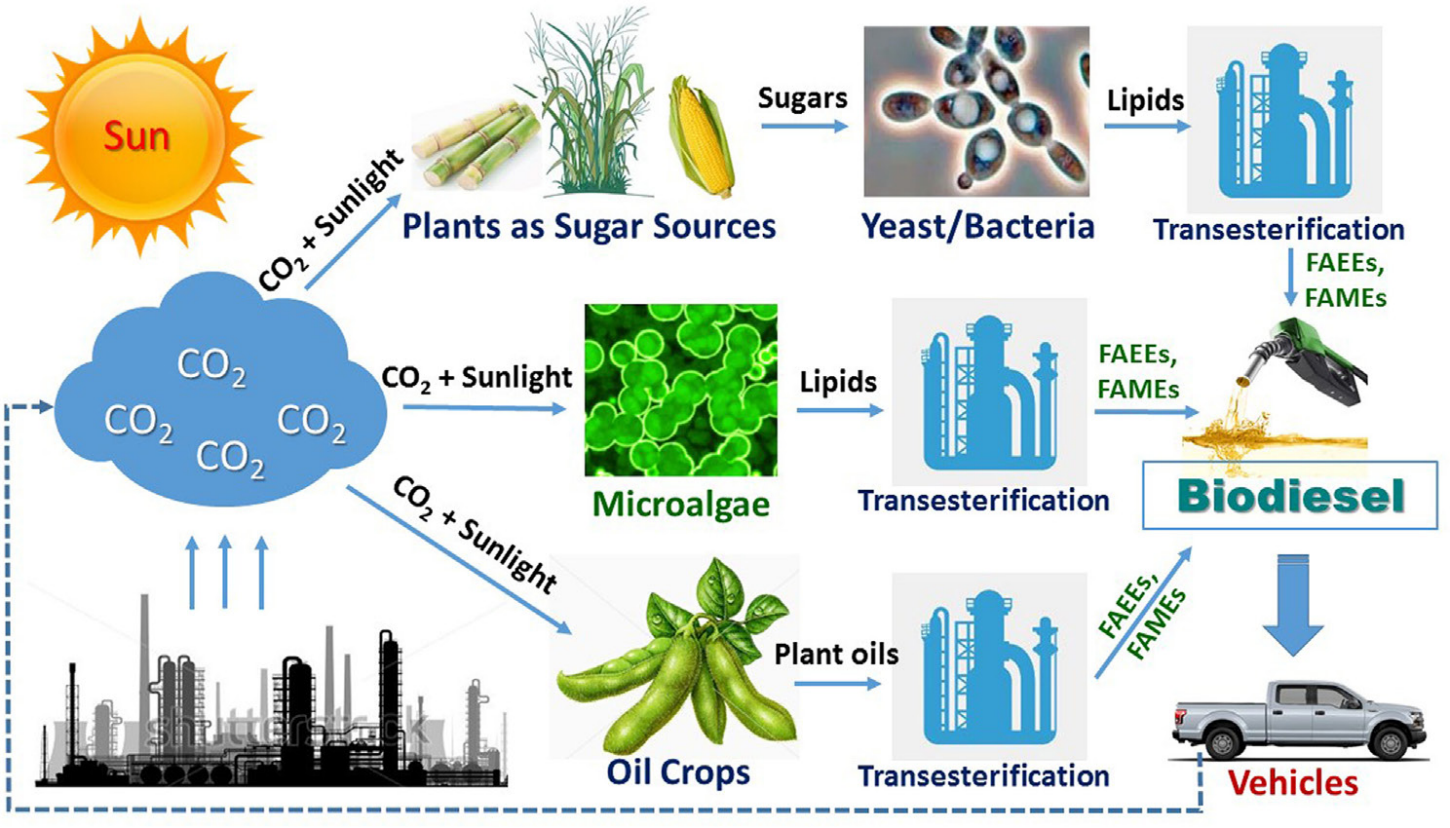
An overview of biodiesel production *via* three typical routes: (1) microbial oil route; (2) microalgae oil route; and (3) plant oil route. Fatty acid ethyl ester (FAEE) and fatty acid methyl ester (FAME) are, respectively, the ethyl and methyl ester of fatty acid.

While bioethanol or biobutanol as the gasoline alternatives are mainly produced by fermentation from sugars derived from starch, sugar cane, or lignocellulosic biomass, biodiesel (fatty acid alkyl esters) is produced by transesterification of oils from microbial cells, microalgae, oil plants, or animal fats by chemical or lipase catalysis (Figure 1). Due to its environmental advantage in significantly lower exhaust emissions of greenhouse gases such as CO, CO_2_, and SO_x_, biodiesel has now attracted more and more attention (Tan et al., 2010). Biobased lipids, produced from plants, microalgae, or microorganisms, should be considered as the most promising, sustainable, and renewable substitute for fossil fuels (Ledesma-Amaro et al., 2015a). For the current time being, plant oils from palm, soybean, corn, and many other oil crops are the major material that is used for biodiesel production (Tan et al., 2010). Due to the concerns of source competition between biofuel and food industries, advanced biofuels including biodiesel are preferred to be produced from non-food biomass or industrial wastes. In the past decades, significant progresses have been made on synthesis of microalgae oils directly from CO_2_ *via* a photosynthesis process (Mata et al., 2010; Rawat et al., 2013; Dickinson et al., 2017). However, the production cost from most current technologies is still too high to be commercialized due to microalgae’s slow growth rate on CO_2_ (typically about 0.1 g DCW/L/d for open ponds and <1 g DCW/L/d for closed photo-bioreactors), inefficiency in large scale photo-bioreactors, lack of efficient contamination control methods for open ponds, and high cost in downstream recovery. The total cost for microalgae biomass are typically from biomass cultivation (60–65%) (Dickinson et al., 2017) and biomass recovery (20–30%) (Mata et al., 2010). Moreover, the locations of the microalgae plants may be limited to only those near power plants with sustainable CO_2_ supply. Due to the current challenges, most companies that originally targeted for microalgae-based biodiesel production currently have either stopped the production or switched to pursue other high-value products that are derived from lipids.

Thanks to the recent advances in both bioprocess and strain engineering, economical sugars become available from biomass containing starch and/or lignocellulose, and then efficiently utilized by oleaginous microorganisms for lipid production. Therefore, microbial oils have several advantages over plant- and microalgae-based oils in terms of cost efficiency and process flexibility. Among all the oleaginous microorganisms, the non-conventional yeast *Yarrowia lipolytica* stands out as an industrial biotechnology platform and has been widely studied for lipid or lipid-related production (Beopoulos et al., 2009; Ledesma-Amaro et al., 2015a, 2016a; Ratledge, 2002, 2004). To produce microbial lipids at a high yield and low cost that may lead to commercial-scale biodiesel production, significant progresses in both cellular and bioprocess engineering in *Y. lipolytica* have been achieved in past decades. For example, it has been demonstrated that a metabolically engineered *Y. lipolytica* can accumulate high lipids, 70–90% of biomass, from glucose only (Liu et al., 2015c; Qiao et al., 2015, 2017). The *Y. lipolytica* yeast was also successfully engineered to directly use starch (Ledesma-Amaro et al., 2015a) or use both C5 and C6 sugars derived from lignocellulosic biomass (Ledesma-Amaro et al., 2016a) for oil production. All these research results suggest *Y. lipolytica* is a promising biocatalyst for commercial biodiesel production in near future.

This review discusses the recent progresses in lipid production by *Y. lipolytica* that may eventually lead to microbial oil-based biodiesel production. The covered technical fields include (1) strain engineering for using various substrates for microbial oil production, (2) metabolic pathway engineering for high-yield fatty acid synthesis (FAS), (3) cell morphology study for efficient substrate uptake and product formation, (4) free fatty acid (FFA) formation and secretion for improved downstream recovery, (5) metabolic engineering for direct biodiesel synthesis, and (6) fermentation engineering for higher volumetric productivities and less operating cost. Considering the price fluctuations in diesel market and to keep the economics of the microbial oils, several examples of production of lipid-related or -derived high-value products are also discussed.

## Strain Engineering for Utilizing Economic Substrates

As previously reviewed by Liu et al. (2015a) and Ledesma-Amaro and Nicaud (2016a), the production costs for bulk chemicals such as biofuels must be minimized so that the bioproduction is competitive to the corresponding petrochemical process. The majority of production cost for fuels and commodity chemicals comes from raw materials, which has to be significantly reduced by using less expensive carbon sources, such as industrial wastes (organic acids, glycerol, oils, and alkanes) and inexpensive sugars derived from biomass (lignocellulosic biomass, starch, inuline, and molasses). However, most well-studied bacteria or yeasts for bioconversions are typically unable to degrade such substrates. Fortunately, advances in synthetic biology and discovery of new enzymes have successfully helped to expand the substrate ranges. In the past decades, strain engineering in *Y. lipolytica* has demonstrated great potentials of using various inexpensive carbon sources to produce biofuels and chemicals. The utilization of diverse substrates by *Y. lipolytica* were also discussed in other reviews (Abghari and Chen, 2014; Liu et al., 2015a; Ledesma-Amaro and Nicaud, 2016a). The major potential substrate candidates, including industrial wastes and biomass-derived sugars, for economical lipid production by *Y. lipolytica* is summarized in Table 1.

**Table 1:** Potential substrate candidates for lipid production by *Yarrowia lipolytica*.

Resources of substrate		Format of substrate being directly used	Key enzymes required for making the substrate	Reference
Industrial wastes	Alkanes	Alkanes	N/A	Thevenieau et al. (2007), Fukuda (2013), Iwama et al. (2014, 2016)
	Plant oils and industrial fats	Free fatty acids + glycerol	Lipase	Aggelis and Sourdis (1997), Papanikolaou et al. (2002a, 2003, 2006), Thevenieau et al. (2010)
	Glycerol from plant oil-based biodiesel process	Glycerol	N/A	Papanikolaou et al. (2002b), Da Silva et al. (2009), Chatzifragkou and Papanikolaou (2012)
	Organic acids	Acetic acid, propionic acids	N/A	Barth and Gaillardin (1996), Venter et al. (2004)

Biomass	Starch	d-Glucose	Amylase	Ledesma-Amaro et al. (2015a)
	Molasses	d-Glucose, d-fructose	Invertase	Nicaud et al. (1989), Lazar et al. (2011, 2013, 2014)
	Lignocellulose	d-Glucose, d-xylose	Cellulase, glucosidase, xylose reductase, xylanase, xylitol dehydrogenase, xylulokinase	Duquesne et al. (2014), Wang et al. (2014), Ledesma-Amaro et al. (2016a), Li and Alper (2016)
	Inuline	d-Fructose	Inulinase	Zhao et al. (2010), Cui et al. (2011), Li et al. (2012)

**Figure 2:**
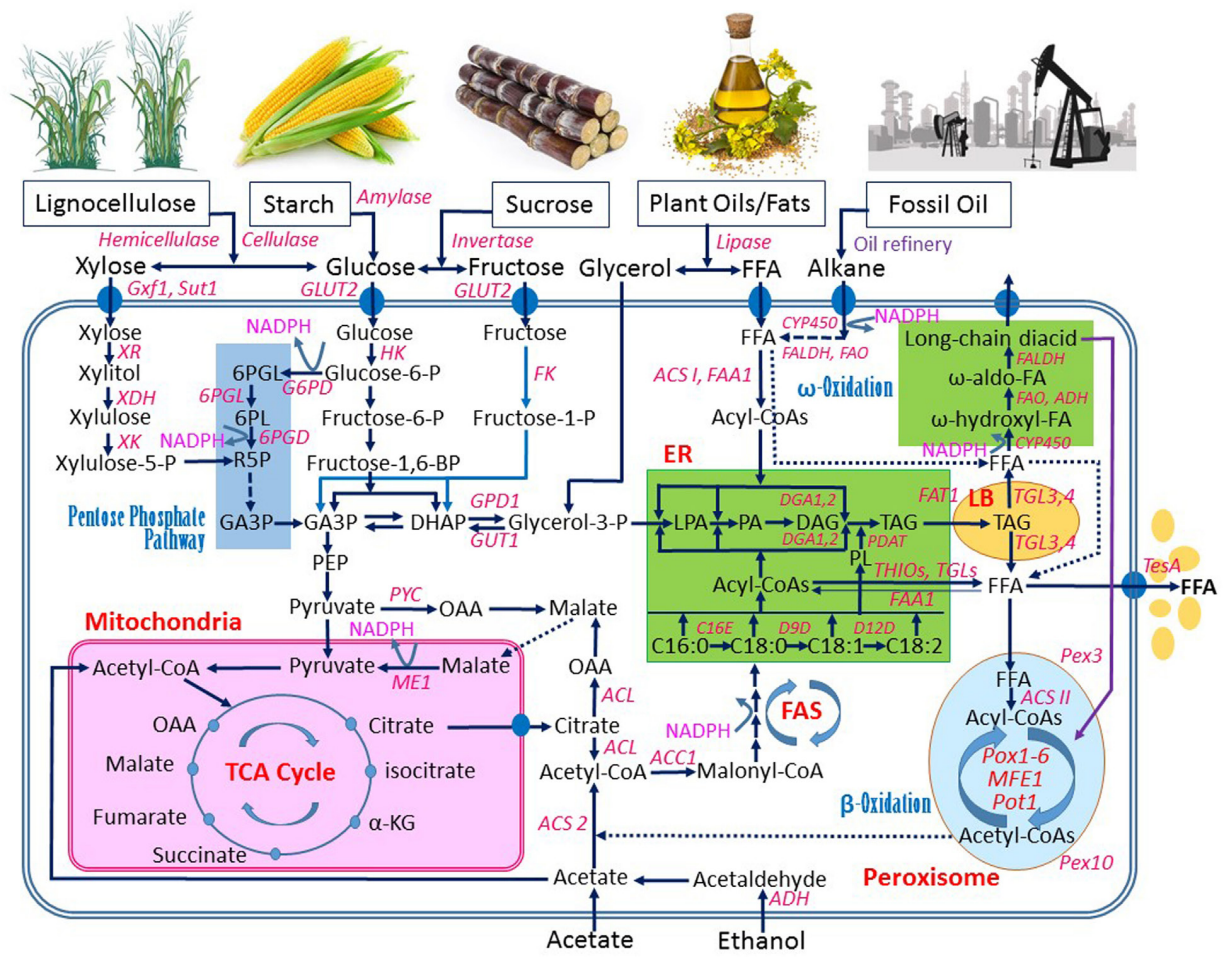
An overview of metabolic pathways in *Yarrowia lipolytica* for synthesis of fatty acids from various substrates. Abbreviations: ACC1, acetyl-CoA carboxylase 1; ACL, ATP citrate lyase; ACS 2, acetyl-CoA synthetase 2; ACS I and ACS II, fatty acyl-CoA synthetase I and II, respectively; ADH, alcohol dehydrogenase; C16E, C16/C18 elongase; D9D, D-9 desaturase; CYP450, cytochromes P450 enzyme; D12, D-12 desaturase; DAG, diacylglycerol; DGA1 and DGA2, DAG acyltransferase; DHAP, dihydroxyacetone phosphate; ER, endoplasmic reticulum; FAA1, fatty acyl-CoA synthetase; FALDH, fatty aldehyde dehydrogenase; FAO, fatty alcohol oxidase; FAS, fatty acid synthase; FAT1, long-chain fatty acid transporter 1; FFAs, free fatty acids; FK, fructose-kinase; G6PD, glucose-6-phosphate dehydrogenase; GA3P, glyceraldehyde-3-phosphate; GLUT2, glucose transporter 2; GPD1, glycerol-3-phosphate dehydrogenase; GUT1 and GUT2, glycerol kinase; GXf1, glucose/xylose facilitator; HK, hexokinase; α-KG, α-ketoglutarate; LB, lipid bodies; LPA, lysophosphatidic acid; ME1, malic enzyme 1; OAA, oxaloacetate; MFE1, peroxisomal multifunctional enzyme 1; PA, phosphatidic acid; PDAT, PL and DAG acyltransferase; PEP, phosphoenolpyruvate; Pex3 and Pex10, peroxisome biogenesis factor 3 and 10; 6PGD, 6-phosphogluconate dehydrogenase; 6PGL enzyme, 6-phosphogluconolactonase; 6PGL intermediate, 6-phosphogluconolactone; PL, phospholipid; Pot1, peroxisomal 3-oxoacyl-CoA thiolase; Pox1~Pox6, acyl-CoA oxidases 1~6; PYC, pyruvate carboxylase; SUT1, sucrose transporter; TAG, triacylglycerol; TCA, tricarboxylic acid cycle; TesA, thioesterase I; TGL3 and TGL4, TAG lipase 3 and 4; THIOs, Acyl-CoA thioesterases; XDH, xylitol dehydrogenase; XK, xylulose kinase; XR, xylose reductase. Filled ovals: transport enzymes. Dash lines: multistep metabolic route. Thin line with arrows: metabolic route with a weaker activity.

Like most microorganisms, *Y. lipolytica* can efficiently use some common sugars, especially glucose, as the substrate for growth and production of various metabolites and enzymes. In addition to utilizing sugars, *Y. lipolytica* is able to grow well on hydrophobic substrates including oils, fats, and alkanes since it is generally isolated from natural environments enriched with these hydrophobic materials (Barth and Gaillardin, 1996). An overview of metabolic engineering of *Y. lipolytica* for FAS from various substrates is shown in Figure 2, which will be used for further discussion in this review.

### Sugar Substrates from Renewable Resources

*Yarrowia lipolytica* can assimilate various C6 sugars such as glucose, fructose, mannose, and galactose though there may be some difference in the specific growth rate and consumption rate for each of the sugars. In an early review conducted by Flores et al. (2000), several non-conventional yeasts including *Y. lipolytica* were compared between their metabolisms on different carbon sources. All these sugar monomers are derived from the biomass containing di- or poly-saccharides. One important task in industrial biotechnology is to find the economical biomass sources that can be efficiently pretreated to provide C5 and C6 sugars.

Glucose is one of the popular carbon sources on market for industrial fermentation. Glucose is generally converted by amylase from starch that is rich in corn, potato, and many other agriculture products. For many fermentation products, especially commodity chemicals and biofuels, glucose is one of the major cost contributors. The total cost can be significantly reduced if the strain can directly metabolize the raw polysaccharide material. For this purpose, Ledesma-Amaro et al. (2015a) expressed both heterologous α-amylase and glucoamylase in the *Y. lipolytica* yeast to decompose the starch to glucose. The engineered strains produced and secreted both enzymes into the culture media, which led to direct growth on starch and produced lipids up to 21% of biomass. Further media optimization with a C/N ratio of 90 improved the lipid content to 27% of biomass.

In addition to using glucose as the sole substrate, the wild-type *Y. lipolytica* can use a mixture of glucose and fructose, but glucose is more preferred than fructose (Lazar et al., 2011, 2013). Lazar et al. (2014) found that hexokinase was critical to fructose uptake, and the strain overexpressing hexokinase grew significantly faster on fructose. Glucose and fructose (inverted sugars) are made from sucrose with the catalysis of invertase. Sucrose is produced from sugar canes or sugar beets, which are rich in South America and the southern region of the United States. Again, the total production cost can be reduced if the yeast can directly use sucrose as the substrate and efficiently convert it into lipids or other high-value products. However, wild-type *Y. lipolytica* cannot directly metabolize it since sucrose needs to be cleaved by an extracellular invertase into glucose and fructose before being used by the yeast. The invertase is encoded by a *SUC2* gene, originally from the *Saccharomyces cerevisiae* yeast. The *SUC2* gene was successfully cloned into *Y. lipolytica* to produce invertase so that the yeast was able to directly metabolize sucrose (Nicaud et al., 1989).

While starch is the major source for making glucose, inulin is an economical polysaccharide for making fructose. As a cold-resistant energy reserve, inulin is a natural carbohydrate source that is present in thousands of plant species, including wheat, onion, and garlic (Niness, 1999). Inulin contains fructose monomer, thus can be used as an important carbon source for microbial fermentation (Pandey et al., 1999). As we discussed earlier, *Y. lipolytica* can grow on sole fructose substrate but not on inulin directly because it lacks inulinase to break it down. To make the strain capable of metabolizing inulin, either the endoinulinase encoded by *EnIA* from *Arthrobacter* sp. *S37* (Li et al., 2012) or the exoinulinase encoded by the *INU1* gene from *Kluyveromyces marxianus* (Liu et al., 2010; Zhao et al., 2010; Cui et al., 2011) were overexpressed. Overexpression of *INU1* in *Y. lipolytica* successfully made the strain able to grow on inulin media, and then converted these substrates into single-cell oils (Zhao et al., 2010) and single cell proteins (Cui et al., 2011). Besides the lipid production, the engineered strain also produced high levels of citrate (Liu et al., 2010). Knockout of the *ACL1* gene and overexpression of the *ICL1* gene further improved citrate production by reducing the followed isocitrate formation in TCA cycle (Liu et al., 2013).

To further reduce the raw material cost of a fermentation product and also to minimize using the potential food source such as starch for the substrate source, lignocellulosic biomass should be considered as the major substrate source for future’s production of biofuels or other fermentation products. The main components in lignocellulosic biomass include cellulose, hemicellulose, and lignin. The hemicellulose contains C5 (xylose) and cellulose contains and C6 (glucose) sugar monomers that are tightly bound to lignin (Davis et al., 2015). The biomass has to be pretreated with acid or base to disconnect the cellulose and hemicellulose from the lignin. Then, the cellulose and hemicellulose are further decomposed to glucose and xylose by cellulase and hemicellulase, respectively (Davis et al., 2015). However, even xylose is successfully extracted from the hemicellulose, wild-type *Y. lipolytica* is unable to consume it. Recently, in both Nicaud and Alper’s research groups, *Y. lipolytica* strains were engineered to metabolize xylose to produce lipids or citric acid (Ledesma-Amaro et al., 2016b; Li and Alper, 2016). Both xylose reductase and xylitol dehydrogenase from *Scheffersomyces stipitis* were first overexpressed in *Y. lipolytica*, but still failed to make good cell growth on xylose. It was found that endogenous xylulokinase (XK) was necessary, and an additional overexpression of XK enabled the strain to grow on xylose at the same rate as the wild-type strain on glucose, which also led to lipid production as well as 80 g/L citrate from xylose. High-lipid production from xylose was achieved by applying the engineered xylose utilization pathway to a high-lipid producing strain, which established a solid base for commercial production of chemicals and fuels from lignocellulosic biomass (Ledesma-Amaro et al., 2016b).

### Hydrophobic Substrates

With hydrophobic cell surfaces, *Y. lipolytica* cells can attach to large lipid droplets in the growth environment and secrete both surfactants and emulsifiers (Mlíčková1 et al., 2004; Thevenieau et al., 2010), which facilitates the first step of hydrophobic substrate utilization. Lipids often exist in the form of triacylglycerol (TAG), which must be broken down into fatty acids and glycerol with the catalysis of extracellular lipases (Thevenieau et al., 2010), so that they can be utilized. *Y. lipolytica* is able to produce and release lipases. The key genes that are involved in fatty acid metabolism have been identified and reviewed (Dulermo et al., 2013, 2015, 2016) and alkanes (Thevenieau et al., 2007; Fukuda, 2013; Iwama et al., 2014, 2016). These genes mainly refer to those encoding the intracellular lipases (Dulermo et al., 2013) and those involved in activation, transport, and degradation of fatty acids or the intermediates in the fatty acid metabolism in the peroxisome (Dulermo et al., 2015, 2016). Understanding the fatty acid metabolic pathway and overexpression of the key genes involved in lipid uptake and degradation are expected to give efficient cell growth on oil substrates and further bioconversion of oil wastes into desired products.

The assimilation and metabolism of hydrophobic materials in *Y. lipolytica* have been previously reviewed by Thevenieau et al. (2010) and Fukuda (2013). Two common hydrophobic substrates, plant oils and alkanes, have been studied to grow *Y. lipolytica* for production of lipids, lipid-derived products, organic acids, and/or other related products. For fatty acid utilization, the transport mechanism is still not well understood. It is believed that selective uptake occurs in *Y. lipolytica* grown on fatty acids with various number of double bonds and chain lengths. Aggelis and Sourdis (1997) found that, when the evening primrose oil was used as the substrate, *Candida lipolytica* consumed the fatty acids in the order γ-linolenic acid (C18:3) > linoleic acid (C18:2) > oleic acid (C18:1) > stearic acid (C18:0). These results were consistent with what was observed by Papanikolaou et al. (2001), in which the unsaturated oleic (C18:1) and linoleic acids (C18:2) were found more easily consumed than the saturated palmitic (C16:0) and stearic (C18:0) acids for cell growth on industrial fats. Fatty acid metabolism is also dependent on the carbon chain length, with two fatty acyl-CoA synthetase (ACS I and II) involved. In the peroxisome, ACS II converts the short-chain fatty acids into fatty acyl-CoAs. In the cytosol, ACS I converts the long-chain fatty acids into fatty acyl-CoAs, which can be used as substrates for lipid synthesis or further transported into the peroxisome for β-oxidation (Kamiryo et al., 1977; Liu et al., 2015a).

The intracellular fatty acids can be degraded *via* both β-oxidation and ω-oxidation pathways (Figure 2). The β-oxidation is taken place in peroxisome. In each β-oxidation cycle, two carbons are lost from the fatty acid backbone and one acetyl-CoA is generated. In *Y. lipolytica*, β-oxidation starts by acyl-CoA oxidases, which are encoded by *POX1*~*6*. The six *POX* genes have different chain-length preferences during the fatty acid degrading process, and the strain with a complete knockout of all *POX* genes cannot degrade fatty acids, which may be used as the strategy to accumulate high-lipid content in biomass (Wang et al., 1999; Mlíčková et al., 2004; Beopoulos et al., 2008). The next two steps in β-oxidation are catalyzed by multifunctional enzyme (MFE). Since MFEs are encoded by a single gene, deletion of *MFE* can give equivalent effect to deletions of all six *POX* genes in terms of stopping β-oxidation for high-lipid production, but the former is technically much simpler to achieve (Dulermo and Nicaud, 2011; Blazeck et al., 2014). The β-oxidation is ended in the last step by the thiolase POT1. Since the β-oxidation takes place in the peroxisome, the very straight-forward strategy to block β-oxidation pathway is to delete the key *PEX* genes to make the peroxisome biogenesis non-functional. For example, deletion of *PEX3* or *PEX10* successfully helped *Y. lipolytica* to achieve high-lipid and omega-3 fatty acid production (Xue et al., 2013; Xie et al., 2015).

Although fatty acids are mainly degraded through β-oxidation in peroxisome, they can also be through ω-oxidation pathway occurring in ER (Gatter et al., 2014). During ω-oxidation, the carbon furthest to carboxylic group of a fatty acid (ω-position) is first oxidized by a fatty acid ω-hydroxylase contained in cytochrome P450 to the corresponding ω-hydroxy fatty acid. Then, the ω-hydroxy fatty acid is converted to the fatty aldehyde, which is catalyzed by fatty alcohol dehydrogenase (ADH). Finally, the fatty aldehyde is converted to the dicarboxylic acid by fatty aldehyde dehydrogenase (FALDH) (Figure 2).

The transport and assimilation mechanism of alkanes in *Y. lipolytica* also remain unclear, though both passive diffusion and active and energy-dependent models were proposed (Liu et al., 2015a). In general, shorter-chain alkanes (C5~C10) cannot be assimilated since they are cytotoxic and may cause a damage in cell membranes (Thevenieau et al., 2007). Therefore, only long carbon-chain alkanes (C10 or above) should be considered for assimilation in *Y. lipolytica*. In recent reviews conducted by Fukuda (2013) and Liu et al. (2015a), three assimilation mechanism steps were proposed: (1) alkanes (C10 and above) are converted into fatty alcohols by the alkane monooxygenase system (AMOS system), (2) the fatty alcohols are converted into fatty aldehydes by long-chain fatty alcohol oxidase (FAO) in the ER, and (3) the fatty aldehydes are converted into the fatty acids by fatty aldehyde dehydrogenase (fatty aldehyde dehydrogenase; FALDH). The enzymes in the last two steps may also be the same as those catalyzing the equivalent steps during the fatty acid ω-oxidation (Gatter et al., 2014). In addition, the fatty alcohols, when transported into ER, are first converted into ω-hydroxy-fatty acids, and then eventually into dicarboxylic acid *via* the ω-oxidation process (Liu et al., 2015a), as also shown in Figure 2.

### Other Hydrophilic Substrates

Glycerol is a major byproduct from plant oil-based biodiesel process. It can be used as an economical substrate for industrial fermentation. *Y. lipolytica* can naturally grow on glycerol with comparable rate as on glucose and is able to produce lipids and organic acids from it (Workman et al., 2013). Since glucose generally represses the uptake of other carbon sources, glycerol may become a preferred carbon source for fermentation (Mori et al., 2013). To improve glycerol utilization, three major metabolic engineering strategies studies have been tried (Ledesma-Amaro and Nicaud, 2016a): (1) facilitating the first catabolism step by overexpression of the glycerol kinase (GUT1); (2) redirecting glycerol toward lipid synthesis and preventing its conversion to DHAP by deletion of G3P dehydrogenase (GUT2) (Beopoulos et al., 2008); and (3) using a glycerol-induced promoter for heterologous expression of glycerol dehydratase (dhaB1) and its reactivator (dhaB2) from *Clostridium butyricum*. These engineering strategies in *Y. lipolytica* significantly increased the production of biomass, lipids, and 2-phenylethanol from glycerol in the fermentation medium (Celinska and Grajek, 2013).

In addition to glycerol, *Y. lipolytica* can use ethanol or acetate as the substrate. *Y. lipolytica* has endogenous functional genes encoding ADHs and aldehyde dehydrogenases that are required to assimilate and ferment ethanol (Liu et al., 2015a). Although high levels of ethanol are toxic to cells, the highest tolerance limit for *Y. lipolytica* in ethanol is up to 3% (v/v) (Barth and Gaillardin, 1996). Also, since glucose represses many functional enzyme activities in *Y. lipolytica*, ethanol may be more preferred for certain products *via* metabolic pathway engineering.

Acetate can also be used as the substrate for *Y. lipolytica* fermentation processes. However, acetate significantly inhibited cell growth when its concentration was higher than 10 g/L, a concentration of 4 g/L or less was well tolerated by the cells (Barth and Gaillardin, 1996). Since only acetate salt, not acetic acid, can be used in fermentation medium to maintain a pH level that is suitable for cell growth, the added acetate, however, causes a pH increase when it is gradually consumed during the fermentation, which leads to inhibition of cell growth when the pH value exceeds the tolerance limit. Therefore, acetate is not recommended as the major or sole substrate. Instead, it is generally co-fed with another substrate. For example, Venter et al. (2004) improved the yield of citrate from *Y. lipolytica* by adding acetate into the sunflower oil medium. In another study, a two-stage fed-batch strategy was developed by Fontanille et al. (2012) to use acetic acid or other volatile fatty acids together with propionic acid to improve cell growth and lipid production.

## Metabolic Engineering for High-Lipid Production

### Fatty Acid Pathway Engineering

In general, fatty acid is synthesized from acetyl-CoA and the cofactor NADPH (Figure 2). This process takes place in the cytoplasm of the cell, and all the reaction steps are catalyzed by the FASs. The glycolytic pathway provides both acetyl-CoA for FFA and glycerol for accumulation of triglycerides (TAG), which is formed by combining one glycerol with three fatty acid molecules. When two of the hydroxyl groups are connected to fatty acids and the third hydroxyl group is phosphorylated with a group such as phosphatidylcholine, a phospholipid is formed, which further forms the lipid bilayers that make up cell membranes. To achieve high-lipid production by metabolic engineering methods, the entire lipid synthesis and formation process needs to be well understood, which includes FAS, TAG formation, remobilization of lipids, transport and activation of fatty acids, and degradation of fatty acids (Ledesma-Amaro and Nicaud, 2016a).

Fatty acid synthesis starts from the building block acetyl-CoA, which is produced through (1) citrate degradation by ATP citrate lyase encoded by *ACL* gene, (2) synthesis from acetate by acetyl-CoA synthetase encoded by *ACS* genes, (3) decarboxylation of pyruvate by a pyruvate dehydrogenase complex (PDC), and (4) degradation of fatty acids through β-oxidation (Vorapreeda et al., 2012). Since oleaginous yeast already has *ACL* genes, no significant improvement in lipid production was seen from overexpression of native or heterologous *ACL* (Dulermo et al., 2015; Ledesma-Amaro et al., 2015b). To initiate the FAS, acetyl-CoA needs to be transformed into malonyl-CoA, which is catalyzed by acetyl-CoA carboxylase (encoded by *ACC1*). Tai and Stephanopoulos (2013) found that overexpression of *ACC1* helped generate a high-lipid production strain. During each FAS cycle, fatty acyl-CoA is formed by FAS from acetyl-CoA (for initiation) and malonyl-CoA (for elongation), therefore, two carbons are added to form a new fatty acid molecule (Ledesma-Amaro and Nicaud, 2016b). NADPH is also consumed during the FAS. In most organisms, the synthesis cycles stop until the fatty acyl-CoAs reach a chain length of 16 or 18 carbons, which are corresponding to C16:0 (palmitic) and C18:0 (stearic) fatty acids. These 16:0 or 18:0 saturated fatty acids can be further elongated and desaturated to make long-chain polyunsaturated fatty acids (PUFAs) (Qiao et al., 2015). For example, C16/C18 elongase (C16E) is responsible for converting C16:0 to C18:0 fatty acids. Desaturases are located in the ER. The Δ9 desaturase (D9D) converts C18:0 (stearic acid) to C18:1 (oleic acid), whereas Δ12desaturase (D12D) catalyzes the conversion of C18:1 (oleic acid) to C18:2 (linoleic acid) (Zhu and Jackson, 2015). The extracellular fatty acids can also be incorporated into the cells, then these FFAs can be activated by fatty acyl-CoA synthetase (FAA1) in cytosol to make fatty acyl-CoAs, which can be further converted into PUFAs by elongases and desaturases (Dulermo et al., 2015).

Once fatty acids are synthesized in *Y. lipolytica*, most of them are stored in lipid bodies, which primarily consist of neutral lipids with about 85% are triacylglycerides (TAGs) and 8% are sterol esters (SE) (Athenstaedt et al., 2006). During the TAG formation process, glycerol generated from the glycolytic pathway is used as the backbone of further synthesis of diacylglycerol (DAG), TAG, and phospholipids. In Kennedy pathway, DAG is converted into TAG either from phospholipid by PDAT (phospholipid:diacylglycerol acyltransferase) or from fatty acyl-CoAs by two DAG acyltransferases (DGA1 and DGA2). There have been many researches reporting that overexpressing *PDAT, DGA1*, and *DGA2* successfully improved lipid production in *Y. lipolytica* (Beopoulos et al., 2012; Xue et al., 2013; Blazeck et al., 2014; Gajdos et al., 2015; Xie et al., 2015).

The structure of lipid bodies is dynamic. On the one hand, more TAGs can be formed and accumulated in the lipid bodies. On the other hand, when the nutrients in the medium are consumed, TAGs can be served as the substrate and degraded into FFAs by two intracellular lipases encoded by *TGL3* and *TGL4*, respectively. The FFAs can be further metabolized through β-oxidation to provide energy for maintenance or additional growth need. Therefore, mutation of the intracellular lipases should help remobilize the produced lipids. Dulermo et al. (2014) found that the deletions of *TGL3* and *TGL4* successfully increased the lipid accumulation by twofold.

For the purpose of high-lipid production, FFAs in cytosol should be converted into TAGs and stored in lipid bodies, as we discussed earlier. Otherwise, they are transported into peroxisome, become activated, and then are degraded into acetyl-CoAs through β-oxidation. Dulermo et al. (2015) proposed a model for fatty acid transport and activation. Basically, FFAs can enter the peroxisome either by unknown transporters in an inactivated form or by the Pxa1/Pxa2 transporters in an activated form. The FFAs in the peroxisome is activated and finally degraded to acetyl-CoAs through the β-oxidation process. To keep high-lipid content, genes involved in β-oxidation such as *POX1-6, MFE1*, and *POT1* or the genes that controls peroxisome biogenesis, including *PEX3* and *PEX10*, should be deleted to avoid or minimize the β-oxidation activities (Dulermo and Nicaud, 2011; Xue et al., 2013; Blazeck et al., 2014; Xie et al., 2015).

Taken together, lipid biosynthesis in *Y. lipolytica* is a complex system that requires an overall optimization of FAS and assimilation, central carbon metabolism, cofactor balance, regulation, and metabolite transport. As addressed by Silverman et al. (2016), the expression of all key genes should be systematically optimized based on specific culture conditions to achieve the highest lipid production. In addition, while optimization of metabolic pathways is the primary focus for most metabolic engineering projects, some fermentation engineering challenges can also be overcome by appropriate strain engineering. For example, *Y. lipolytica* requires efficient oxygen transfer in a bioreactor for both cell growth and lipid production. However, the strain may also suffer high-oxidative stress under the aerobic bioreactor conditions. Xu et al. (2017) recently reported that maintaining redox homeostasis and detoxifying reactive aldehydes were helpful to overcome the oxidative stress, and then improve cell fitness and lipid production.

### Selecting Appropriate Promotors for Gene Expression

To successfully engineer the FAS pathway, promoters of *Y. lipolytica* genes are extremely important to control the expression levels (Xie et al., 2017b). A set of promoters, with similar or higher strength than the *TEF* promoter, were isolated from genes involved in central metabolism pathways (Muller et al., 1998). The β-glucuronidase (GUS) reporter was elected to express under each individual promoter, and the signal strength was determined by using quantitative fluorometric assays, which was used for comparison (Jefferson et al., 1987). It was found that the *FBAin* was the strongest promoter among all tested. In addition, the *GPM1, GPD1*, and *FBAin* promoters were 1.0, 2.5, and 5.0 times as strong as the *TEF* promoter, respectively (Hong et al., 2011). Interestingly, the activity of a nitrogen-sensitive promoter *YAT1* increased by 35-fold when the fermentation medium was changed from nitrogen-rich to nitrogen-limiting conditions (Xue and Zhu, 2012). This unique feature provides an advantage for application of *YAT1* in oleaginous yeast which typically accumulates lipids under nitrogen-limiting conditions (Xie et al., 2017b). Based on the results, the strength of all the promoters examined should be in the following order: *FBAin* > *YAT1* > *FBA* > *GPD, EXP* > *GPAT* > *GPM* = *TEF*.

To further increase the promoter diversity, Alper et al. (2005) generated a new *Y. lipolytica* promoter pool by random mutagenesis, which provides many more options for gene expression control in the yeast. Later, Blazeck et al. (2011) further expanded and generated a high-efficiency, tunable promoter pool, with each promoter consisting of an enhancer element and a core promoter element. By this approach, the strongest promoters for *Y. lipolytica* at that time were created. In their recent research, a more generalized strategy was developed, which was able to identify both novel upstream activating sequences and core promoters for the *de novo* construction of strong, synthetic hybrid promoter libraries (Blazeck et al., 2013b). This big effort led to a new promoter nearly eight times as strong as the TEF promoter. In addition to the strong and constitutive promoters, inducible promoters are desired for many *Y. lipolytica* fermentation processes so that the gene expression levels during cell growth and production phase can be tunable and regulated to achieve the best productivity. Trassaert et al. (2017) isolated, characterized, and modified the promoter of the EYK1 gene (pEYK1), which is repressed by glucose and glycerol, but strongly induced by erythritol and erythrulose. Further deletion of the *EYK1* gene allowed the use of erythritol and erythrulose as free inducer for efficient heterologous protein production.

### Genome Editing Technology

In terms of genetic operation of *Y. lipolytica*, all the genetic modifications are suggested to be performed in chromosomes to obtain a high-genetic stability of the strain, which is very important to commercial-scale fermentation processes (Xie et al., 2015, 2017b). When a target gene is to be inserted into chromosome, the non-homologous end-joining (NHEJ) is much more frequent than homologous recombination (HR) in *Y. lipolytica* (Kretzschmar et al., 2013; Verbeke et al., 2013). Therefore, it is more difficult to insert a target gene, especially with a big size, into a specific site. The DNA-binding protein KU70 or KU80 plays a key role in NHEJ. It was found that deletion of *ku70* significantly hinders NHEJ efficiency and improves HR, which can be applied to targeted gene insertion in *Y. lipolytica* (Gao et al., 2016; Schwartz et al., 2016).

Recently, the CRISPR-Cas9 technology has demonstrated revolutionary genome editing, from microbial to more advanced plant and animal cells. Gao et al. (2016) described how a CRISPR-Cas9 system was constructed in *Y. lipolytica* for genome editing. In their study, Cas9 and relevant guide RNA expression cassettes to target gene were transported by using a single plasmid (pCAS1yl or pCAS2yl). Within only 4 days, two Cas9 target genes were repaired by NHEJ or HR with a highest efficiency of 86% for the native *Y. lipolytica* and 94% for the k*u70*/*ku80* double-knockout strain, respectively. Multigene editing was also investigated by NHEJ with pCAS1yl. An efficiency of 37% for double mutation and 19% for triple mutation were achieved. In the recent study conducted by Schwartz et al. (2016), much higher efficiencies (92% or higher) were achieved for single gene disruption in which single guide RNAs (sgRNA) were transcribed with synthetic hybrid promoters. When both Cas9 and sgRNA expressing plasmid were cotransformed, a HR efficiency of 64% was achieved. Disruption of NHEJ led to 100% HR efficiency. Most recently, Schwartz et al. (2017) further developed a CRISPR interference (CRISPRi) system for gene repression in *Y. lipolytica*. Up to 90% HR efficiency was achieved by only repressing *ku*70/80 for NHEJ instead of permanently knocking it out.

### Increasing NADPH Availability

In addition to acetyl-CoA, NADPH is necessary for the FAS. Qiao et al. (2017) revealed that 16 mol of NADPH are required to synthesize 1 mol of stearic acid (C18:0). NADPH is mainly generated *via* the pentose phosphate pathway (Tang et al., 2015; Wasylenko et al., 2015; Zhao et al., 2015). In addition, the reaction of the decarboxylation of malate to pyruvate, catalyzed by the malic enzyme, is believed another important NADPH source (Figure 2). However, Zhang et al. (2013) reported that no significant improvement in NADPH regeneration was seen when malic enzyme was overexpressed in *Y. lipolytica*, alternative routes should be examined.

So far, even the best yields of microbial lipids obtained are insufficient for commercial production. Since NADPH is one of limiting factor in FAS, it was suggested that the FA conversion yield be improved by capture of most electrons generated from substrate catabolism (Qiao et al., 2017). A strategy of achieving this goal is to convert NADH to NADPH, which was investigated by Qiao et al. (2017) in 13 engineered strains of *Y. lipolytica*. In bioreactor experiments, the best engineered strain produced lipids at a productivity of 1.2 g/L/h and a conversion yield of 0.27 g FAMEs/g glucose, which improved the yield by 25% over previously engineered yeast strains (Tai and Stephanopoulos, 2013; Qiao et al., 2015). Interestingly, the yield improvement did not cost any extra energy. For example, oxygen uptake rate of the best lipid producer was reduced due to the decreased NADH oxidization activities. The results showed that redox engineering should be the important direction that may eventually lead to commercially viable lipid production. In addition, the achieved titer, yield, and productivity of fatty acid methyl esters were close to fulfilling the requirements (90 g/L, 1.3 g/L/h, and 0.28 g-fatty acids/g-lignocellulosic sugars) determined by the National Renewable Energy Laboratory to support the US DoE’s 2017 cost goal, $5/gallon gasoline equivalent (Davis et al., 2015).

### Other Metabolic Engineering Strategies

In addition to all the strategies discussed earlier, some other efforts have also been taken to further improve lipid production by *Y. lipolytica*. For example, regulators are important for oleaginous phenotype engineering. In general, *Y. lipolytica* requires nitrogen to build biomass in growth phase but also needs nitrogen limitation or starvation for lipid production in oleaginous phase (Xie et al., 2015). Therefore, there might exist a global regulator to balance cell growth, lipid production, and the transition between the two phases. Seip et al. (2013) found that SNF1 pathway played an important role in the switch from growth to oleaginous phase. Mutation of *snf1* gene in *Y. lipolytica* led to a much earlier lipid production and significantly improved the overall omega-3 EPA production by 52% for one of the early-stage omega-3 strains in DuPont. Liu et al. (2015b) also identified and validated a mutant mga2 protein that regulates the desaturation and potent lipogenesis in *Y. lipolytica*. The mga2 mutant strain was able to produce significantly more unsaturated fatty acids. In addition, combination of mga2 mutant with other metabolic engineering efforts was expected to produce 25 g/L lipids. Wang et al. (2013) revealed that disruption of the *MIG*1 gene significantly enhanced the transcript levels of many genes involved in lipid biosynthesis. The *MIG*1 mutation in *Y. lipolytica* improved the lipid content from 36 to 49% in biomass.

Despite the significant progresses so far in understanding the lipid biosynthesis in *Y. lipolytica*, many key genes, proteins, and pathways involved in the lipid production have still not been identified. Omics-based studies such as genomics, proteomics, and metabolomics are important for understanding lipogenesis and further engineering in *Y. lipolytica*. For example, Pomraning et al. (2016) investigated the metabolome, proteome, and phosphoproteome data that were related to lipid accumulation under nitrogen limited conditions. Many key genes involved in TCA cycle and FAS were upregulated and in β-oxidation were downregulated. Thus, excess citrate and some other TCA cycle intermediates were produced as result of nitrogen limitation, which then lead to twofold increase in acetyl-CoA, the precursor for starting the fatty synthesis. Also, the decreased capacity for β-oxidation led to lipid accumulation at high levels. Recently, Kerkhoven et al. (2017) also used the multi-omics research and discovered that leucine biosynthesis plays a key role in regulating the lipid accumulation in *Y. lipolytica*.

## Special Cellular Engineering for FFA or Biodiesel Production

### Secretion of Produced FFAs

To make the microbial lipid-based biodiesel economically viable, the total lipid titer (g lipid/L volume), productivity (g lipid/L/h), and conversion yield (g lipid/g substrate) in the fermentation need to be high enough. In addition, the produced lipids should be easily separated from the fermentation medium and recovered so that the downstream process cost is minimized. One potential solution to all these challenges is to make the *Y. lipolytica* to secret the produced fatty acids into the medium (Ledesma-Amaro et al., 2016b). Comparing to the intracellular lipid production, secretion of fatty acids into medium will have three advantages: (1) the specific fatty acid production (g fatty acids/g biomass) in a format of secreted FFAs can be significantly higher than in the format of intracellular lipids since the physical boundary (cell membrane) that limits the lipid content in a cell no longer exists; (2) the secreted FFAs can be easily separated and recovered at minimal cost since they are insoluble in aqueous medium; and (3) cells are well mixed in aqueous medium while the secreted fatty acids can stay on top of the medium without affecting fermentation performance. However, this may be a challenge for cells with high content of intracellular lipids since high-lipid content makes cells lighter than aqueous medium (Liu et al., 2015c), which makes those cells more difficult to be well mixed for efficient fermentation, especially for the fermentation in a commercial-scale bioreactor.

Intracellular lipid production by yeasts and bacteria have been widely studied, but secretion of fatty acids has not been well investigated, with very few examples reported for the classical yeast *S. cerevisiae* (Ledesma-Amaro et al., 2016b). Some yeasts such as *Starmerella bombicola* can naturally secret lipid-derived molecules and sophorolipids (Van Bogaert et al., 2007). Baker yeast was also engineered to promote FFA production and secretion by disruption of long-chain acyl-CoA synthetases (*scFAA1* and *scFAA4*) (Scharnewski et al., 2008; Li X et al., 2014). Thioesterases are important for secreting the produced FFAs. For example, the strain overexpressing *TesA* from the *Escherichia coli* produced 207 mg/L FFA (Chen et al., 2014). The FFA production was improved to 520 mg/L by further expression of a truncated version of the *Mus musculus* ACOT5 (Runguphan and Keasling, 2014). To further improve the extracellular FFA production, multiple genes involved in acyl-CoA synthesis (Δ*faa1*, Δ*faa4*, and Δ*fat1*) and β-oxidation (Δ*faa2*, Δ*pxa1*, and Δ*pox1*) were disrupted by Leber et al. (2015). Together with the strategies of enhancing neutral lipids and releasing FFA, 2.2 g/L FFA was produced by the engineered *S. cerevisiae* in shake flask experiments, and a conversion yield of 0.11 g FFA/g glucose was achieved (Leber et al., 2015). Though the titer and yield of the secreted FFA production were still far from commercial target values, the research data showed great potentials if the more advanced oleaginous organisms such as *Y. lipolytica* are used for secreted FFA production.

Ledesma-Amaro et al. (2016b) described their first study aimed to secrete fatty acids in *Y. lipolytica* by two different rational designs. The first strategy was to enhance the TAG synthesis, lipid body (LB) formation, and LB remobilization so that FFAs in a background were not degraded. The second strategy was to mimic bacteria by disruption of LB formation and to produce FFAs by novel and re-localized thioesterases. To further improve FFA production and recovery yield, an *in situ* product recovery process with an organic phase was developed. More than 20 g/L total lipids (intracellular + extracellular) was produced from about 17 g/L biomass in a 200-mL bioreactor. The total lipids produced was more than 120% of the biomass, which suggests that lipid production, by secreting out of cells, could exceed the limit even with the physical boundary of the cells. Combining this technology with other breakthroughs in fermentation process development may lead to economically viable lipid or lipid-based biodiesel production.

### Direct Production of Biodiesels by Fermentation

Engineering for secretion of produced fatty acids makes the microbial oil-based biodiesel more economically viable, as discussed earlier. However, the most ideal process would be direct production of biodiesel from fermentation, which needs to be developed with this yeast (Abghari and Chen, 2014). Fortunately, there has already been some early studies on using recombinant *E. coli* and *S. cerevisiae* for direct production of fatty acid ethyl esters (FAEEs) or methyl esters (FAMEs), which could be used for further exploration in *Y. lipolytica*.

To synthesize FAEEs or FAMEs, the metabolic engineering strategies include the general pathway engineering and optimizations, not only for FAS but also for ethanol or methanol biosynthesis. Due to relative simplicity of the metabolic pathways and the availability of various genetic tools, *E. coli* was the first microorganism studied for direct production of both FAEEs and FAMEs. In addition, FAEEs within a concentration of 100 g/L were non-toxic to *E. coli* cells (Steen et al., 2010). Kalscheuer et al. (2006) investigated the heterologous expression of a PDC, an ADH, and an unspecific acyltransferase in *E. coli* to achieve the biosynthesis of FAEEs. Using this strategy, ethanol was esterified with subsequent fatty acids inside the cell to make FAEEs, with majority being ethyl oleate. Up to 1.28 g/L FAEE at 26% of the biomass was achieved in a 2-L fed-batch fermentation. Steen et al. (2010) also demonstrated that tailored fatty esters as biodiesel were produced by successfully engineering the *E. coli*. This FAEE biosynthetic pathway was added into an ethanol-producing strain expressing the *PDC* and *ADH2* from *Z. mobilis*, which led to the production of 0.7 g/L FAEEs. Furthermore, the *E. coli* strain was also engineered to produce FAEEs directly from hemicellulose by expressing hemicellulases. To significantly improve the titer and productivity of the produced FAEEs, Elbahloul and Steinbuechel (2010) used an engineered *E. coli* harboring a p(Microdiesel) plasmid for fed-batch production of FAEEs at pilot scale (16–20 L). The two-stage fermentation was conducted by using glycerol for cell growth, and then followed by feeding glucose and oleic acid for FAEE production. About 61 g/L biomass containing 25% FAEEs was produced in the fermentation. In addition to production of FAEEs, FAME production was also investigated by Nawabi et al. (2011) by manipulating the methionine biosynthesis and expressing methyltransferase and thioesterases in *E. coli*. Unfortunately, only 0.016 g/L FAMEs were obtained, which indicates it is more challenging for biosynthesis of FAMEs to be considered for practical biodiesel production.

The classic yeast *S. cerevisiae* was also examined as a host strain for production of FAEEs. In the early research conducted by de Jong et al. (2014), a *S. cerevisiae* strain was used to produce FAEEs from ethanol and fatty acyl-CoAs by heterologous expression of a wax ester synthase (WS2). The strain was engineered in the central metabolism to provide enough acetyl-CoA and NADPH for production of acyl-CoAs, then it was further evaluated for FAEE production. Both ethanol degradation and the phosphoketolase pathways improved the FAEE yield. However, only about 10 mg/L FAEEs were produced in the flask experiments (with only 0.5% FAEE in DCW). The specific FAEE production was 50-fold lower than what has been achieved by the metabolically engineered *E. coli* strains. Since *S. cerevisiae* yeast is not a typical fatty acid producer, it would be still interesting to examine the direct FAEE production by using the oleaginous yeast *Y. lipolytica* in future.

In addition to ethyl or methyl esters as a direct diesel format, other types of oleochemicals such as fatty alcohols and alkanes can also be considered for direct diesel or diesel-like applications. Xu et al. (2016) recently examined new pathway engineering strategies in *Y. lipolytica* to establish a platform for sustainable production of fatty ethyl esters, fatty alcohols, and/or fatty alkanes. For the best engineered *Y. lipolytica* strain, 142.5 mg/L FAEEs, 23 mg/L fatty alkanes, 2.2 g/L fatty alcohols, 9.7 g/L FFAs (with 36% C12~C14), and 66 g/L TAGs were produced in a 3-L fed-batch bioreactor. Blazeck et al. (2013a) also successfully introduced a soybean lipoxygenase in *Y. lipolytica* to cleave linoleic acid (C18:2) to pentane (C_5_H_12_). However, the poor titer achieved (4.9 mg/L) suggests that more significant breakthroughs in both strain and fermentation engineering should be achieved before it could be considered for a potential application.

### Cell Morphology Engineering for Efficient Substrate Uptake and Product Secretion

*Yarrowia lipolytica* is a dimorphic yeast, which exhibits different cell morphology as the growth environment changes, from typical round yeast cells to mycelium shape (Pérez-Campo and Domínguez, 2001). Domínguez et al. (2000) found that both environmental conditions and genetic regulatory mechanism play important roles during the transition of *Y. lipolytica* morphology. In many cases, *Y. lipolytica* cell morphology is transitioned from round, yeast-like shape to elongated filament shape, hyphae or mycelia, under stressful environmental conditions such as high-osmotic pressure, nutrient limitation, and other non-preferred growth conditions. In some other cases, this transition is more related to the excretion of produced products. For example, Wucherpfennig et al. (2011) found that morphology change of *Aspergillus niger* to elongated shape significantly improved the specific productivity of fructofuranosidase. The morphology change of *Y. lipolytica* cells may help to increase the specific surface area to reach more nutrients in the environment or secret the product. Although the *Y. lipolytica* morphology may subject to self-transition over new environmental condition changes, the obtained morphology status may not be steadily maintained for the best substrate uptake and product secretion. Ideally, the genes that relates to the yeast morphology can be identified and modified so that a desired morphology will be obtained to benefit the fatty acid production.

The first strategy is to control the morphology by identifying and modifying the native genes in *Y. lipolytica*. To understand the proteome relating to the morphology transitions of the *Y. lipolytica* cells, proteome of the wild-type and mutant *Y. lipolytica* was analyzed by Morín et al. (2007) during the yeast-to-hyphae transition. A 2-D gel electrophoresis and mass spectrometry method was used and some of the key proteins related to morphology transition were identified. Later, three Ras proteins (YlRas1p, YlRas2p, and YlRas3p) that are critical for dimorphic transition in *Y. lipolytica* were identified by Li et al. (2014a). It was further confirmed that the major protein regulating the dimorphic transition is YlRas2p. Li et al. (2014c) found that another important protein in regulating the morphogenesis of *Y. lipolytica* was Rap GTPase (YlRsr1p). All these results provided us an initial clue for stable morphology control of *Y. lipolytica* by genetic modification.

The second strategy is to introduce a heterologous expression of some bacteria genes in *Y. lipolytica* for morphology control. The experience of genetic modification in bacteria for cell morphology control can also be adopted in *Y. lipolytica* yeast. For example, a type of cytoskeletal filament bundle MreB was found critical to control bacteria’s morphology and determine the cell shape as either round or rod-like (Jones et al., 2001, Cabeen and Jacobs-Wagner, 2005, Jiang et al., 2011). Bacteria cell wall is mechanically reinforced by MreB to keep a stable morphology. Crescentin in *Caulobacter crescentus* is similar to MreB. When crescentin was deleted, the cells lost their curved shapes, and transitioned to straight rods (Ausmees et al., 2003). All these studies will provide additional opportunities of re-structuring *Y. lipolytica* shape by genetic modifications, which may lead to further improvements in substrate uptake and/or fatty acid production.

## Fermentation Process Engineering

### High-Throughput Strain Screening by Fermentation

After new strains are generated, a series of fermentation engineering steps, including strain screening, fermentation optimization, and process scale-up, are required before a large-scale production can be carried out (Xie et al., 2015, 2017b). Micro- or mini-scale bioreactors are typical small bioreactor system employed for strain screening, which usually have very small working volumes, from 1 to 100 mL, so that dozens of candidate strains can be tested at one time. For the small bioreactors to achieve consistent performance as seen in large scale bioreactors, each small bioreactor vessel must have high-quality process controls (Xie et al., 2017b). Many small bioreactor tools are now available for screening of candidate strains or process conditions, these include the multi-well blocks (Danielson et al., 2004), test tubes (Stockmann et al., 2003), shake flasks (Anderlei and Büchs, 2001; Büchs, 2001), and microtiter plates/bioreactors (Duetz et al., 2000; Amanullah et al., 2010). Unfortunately, the major challenge for most small-scale bioreactors is the lack of precise control system, especially for pH values and DO levels. In addition, too small reactor size for some of those bioreactor systems (e.g., less than 5 mL) also limits the application in screening of lipid-producing strains since the minimal sample size required for biomass and lipid titer analysis should be a few milliliters. Recently, a Micro-24 Bioreactor system, which independently controls T, pH, and DO (pO_2_) in each of the 24 reactors, was used for omega-3 fatty acid strain screening (Xie et al., 2017b). A detailed method was also described earlier (Xie, 2012). By using this system, both online and offline data, including T, pH, DO (pO_2_), biomass concentrations (DCW), lipid titer and yield, and byproduct concentrations, were all available, which successfully helped the scale-up of the selected strain and process from micro-scale (a few milliliters) to commercial-scale (hundred cubic meters) bioreactors (Xie et al., 2017b).

### Optimization of Batch and Fed-Batch Fermentation

High-lipid production should be achieved in a bioreactor with significantly higher cell density to reduce the production cost. Under bioreactor conditions, acetyl-CoA and NADPH for FAS has to be assured under nutrient limited but carbon excess conditions (Ochsenreither et al., 2016). The continuous production of acetyl-CoA is achieved by a cascade of enzyme reactions triggered by a nutrient limitation (usually nitrogen limitation) leading essentially to a citrate accumulation in the mitochondria. Under a nitrogen-limited condition, the activity of AMP deaminase is increased considerably, which catalyzes the cleavage of AMP to IMP and ammonia. The AMP decrease halts the activity of isocitrate dehydrogenase, which catalyze the conversion of isocitrate into α-ketoglutarate in the TCA cycle. Therefore, isocitrate is converted back to citrate by the enzyme aconitase, which leads to citrate acid accumulation. The formed citric acid is transported to cytosol, further converted to acetyl-CoA by ACL, and eventually goes to the FAS pathway (Botham and Ratledge, 1979; Boulton and Ratledge, 1981a,b; Evans and Ratledge, 1984, 1985; Wynn et al., 2001; Ratledge, 2002). If fatty acid pathway is not efficient, citrate is produced as a major byproduct.

Based on the mechanism of citrate formation, which is the key step to introduce carbon flow to FAS, the fermentation for lipid production by *Y. lipolytica* should be a two-stage fermentation process (Xie et al., 2015, 2017a,b). In the first stage (cell growth), the *Yarrowia* cells are grown in a medium containing both carbon source and nitrogen source. Nitrogen was pre-charged in the medium in a format of organic source (yeast extract or corn steep liquor) and inorganic source [(NH_4_)_2_SO_4_ or other ammonium salts]. The inorganic nitrogen can also be provided in the form of NH_4_OH by using it for pH control in the first stage (Xie et al., 2015). The second stage starts when the residual nitrogen from the first stage is depleted, then cell growth stops and the lipids start to accumulate. Under nitrogen limited or starved conditions, *Yarrowia* cells continuously convert the added substrate into lipids. In terms of other fermentation conditions, the temperature is typically controlled within 28–30°C, and the pH value is within 5.5–7.0 (Qiao et al., 2017; Xie et al., 2017a). *Y. lipolytica* requires oxygen for both cell growth and lipid formation, a typical dissolved oxygen level of 20% air saturation or above is suggested (Qiao et al., 2017; Xie et al., 2017a).

To reduce the intensive experimental work and accelerate the fermentation process development, dynamic mathematical models were established based on the experimental data and the understandings of the key metabolic activities in the strain and the process characteristics. The model equations were used to help analyze, predict, and optimize the fermentation performance under various conditions without the need of labor-intensive experiments (Xie et al., 2015, 2017a). In the omega-3 fatty acid project, the fermentation model was successfully built and used to predict the titer, rate, and yield of lipids, and further guided the process optimization and scale-up (Xie et al., 2015, 2017a). Due to the common features of the strain and fermentation process, this model can be further modified and extended to any other lipid production processes that uses *Y. lipolytica* under fed-batch and continuous bioreactor conditions.

### Continuous Fermentation for Lipid Production

Most industrial fermentation processes are batch or fed-batch fermentation operations due to their advantages of simpler operation, lower contamination risks, higher product titer, and available knowledge and experience in scale-up, which were mainly developed in the middle of last century (Dombek and Ingram, 1987; Xie et al., 2001). However, batch and fed-batch processes usually have significantly lower volumetric productivities when compared with most chemical processes that employs continuous operations. Continuous biomanufacturing processes have attracted more and more attention as a means to improve volumetric productivities (Taylor et al., 1995; Menzel et al., 1997; Ni and Sun, 2009; Li et al., 2011). The productivities in most reported continuous fermentation processes were improved, but typically at the expense of product concentration, conversion yield, or both (Mutschlechner et al., 2000; Ethier et al., 2011).

**Figure 3:**
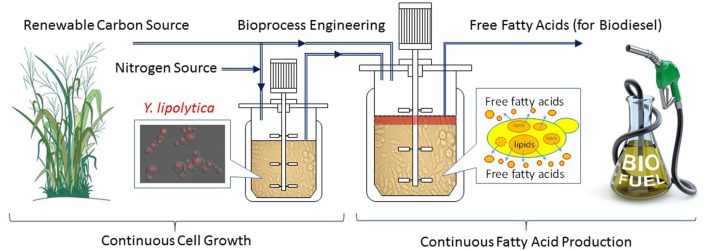
A conceptual continuous fatty acid-based biodiesel production from renewable resources by metabolically engineered *Yarrowia lipolytica*.

Continuous fermentation can be either single-stage or multi-stage depending on target molecule production kinetics (Xie et al., 2017a). Single-stage continuous fermentation is suitable for growth associated bio-production processes (Shuler and Kargi, 2002). However, production of fatty acids is believed non-growth associated. Continuous processes for non-growth-associated kinetics typically employ multiple fermenters to stage the fermentation process into discreet segments such that the optimal conditions for cell growth and product formation can be achieved in separate fermentors. The biggest challenge for a continuous process is to achieve significantly higher productivities without sacrificing product titers and/or conversion yields (Gapes et al., 1996; Mutschlechner et al., 2000; Chang et al., 2011; Koller and Braunegg, 2015). In our recent research (Xie et al., 2017a), a two stage continuous fermentation process for omega-3 EPA (C20:5) production by a recombinant *Y. lipolytica* was developed, based on the model simulation results of various bioreactor operations (fed-batch vs. single-stage continuous vs. two-stage continuous). The novel continuous process consisted of a small growth tank (Stage 1) and a large production tank (Stage 2), with each controlled on its own optimal conditions. Compared with the optimized fed-batch process, the two-stage continuous experiments successfully improved the volumetric lipid productivities by 80% and the lipid concentrations by 40% while maintained similar conversion yields. In addition, the engineered *Y. lipolytica* strain was found slowly evolved to redirect the byproduct formation to more lipid production during the 6-week continuous experiment. The possible reason was that the byproduct, mainly citric acid, is growth inhibitive, therefore the evolution toward reducing citrate and re-introducing the carbon flow to FAS became more favorite to cell growth. Due to the success of the two-stage continuous fermentation in omega-3 fatty acid production, this novel process can be adopted for efficient biodiesel production by combining the strain engineering progress in secretion of fatty acids (see the previous section in special cellular engineering). Figure 3 shows a conceptual continuous fatty acid-based biodiesel production from renewable resources such as lignocellulosic biomass. The combination of all innovations in fermentation process engineering (Xie et al., 2017a) and the strain engineering for secreted fatty acid production (Ledesma-Amaro et al., 2016b) will make the continuous biodiesel production process more technically attractive and cost-effective.

## Lipid-Related or -Derived High-Value Products

Although microbial oil-based biodiesel is a very promising direction to replace fossil fuels in future, the fluctuations and the uncertainty of the fuel market require the technology platform have a flexibility for more product options. For this purpose, high-value products derived from lipids or related to lipids should be considered to provide more economical biomanufacturing options. Here, two product examples will be discussed: omega-3 fatty acids and carotenoids.

The two major omega-3 fatty acids on the market are eicosapentaenoic acid (C20:5; EPA) and docosahexaenoic acid (C22:6, DHA), which are believed critical in improving human’s heart health, immune function, mental health, and infant cognitive development (Xie et al., 2015). Currently, the major source for omega-3 fatty acids is from fish oil, but its availability and sustainability are questioned due to the increased concerns of overfishing and unpredictable ocean contaminations. To produce DHA, Martek (now part of DSM) developed a large-scale microalgae fermentation technology (Kyle, 2001). To produce EPA, DuPont used a metabolically engineered *Y. lipolytica* to provide a sustainable, land-based source of EPA (Xie et al., 2015). Specifically, the Δ9/Δ8 pathway was introduced into the yeast by overexpressing desaturases and elongases to synthesize omega-3 EPA (C20:5). The metabolic pathways for most byproducts were blocked and the FAS pathway was optimized by carefully controlling and balancing expression levels of key pathway enzymes (Xue et al., 2013; Xie et al., 2015). It was also found that disruption of peroxisome biogenesis by knockout of *PEX10* gene led to significant increase in production of EPA and the total lipids (Xue et al., 2013). The result of the metabolic engineering effort led to two commercial omega-3 products to DuPont (Xie et al., 2015).

Carotenoids including lycopene, β-carotene, canthaxanthin, and astaxanthin are another type of high-value products that attract attention from biotechnology industry due to their health benefits for both humans and animals (Zhu and Jackson, 2015) and the great market opportunity, estimated to reach $1.4 billion in 2018 (Ye and Bhatia, 2012; Lin et al., 2014). Currently, the majority of carotenoids is chemically synthesized or sourced from some natural organisms. To meet the increased need, much effort has been taken to metabolically engineer *E. coli, S. cerevisiae*, and *Y. lipolytica* to produce carotenoids (Ye and Bhatia, 2012; Lin et al., 2014; Gao et al., 2017). Since carotenoids are hydrophobic and only soluble in oil phase, strains that can accumulate high lipids are preferred as the host for efficient carotenoid production so that the lipid bodies can serve as the storage space for carotenoids. Synthesis of carotenoids starts from two C5 precursors, isopentenyl diphosphate (IPP) and dimethylallyl diphosphate (DMAPP), which are synthesized from acetyl-CoA by HMG reductase *via* the mevalonate pathway in *Y. lipolytica*. IPP and DMAPP are further converted into geranyl pyrophosphate (C10), farnesyl pyrophosphate (C15), and geranylgeranyl pyrophosphate (C20) in a series steps before they are eventually converted to various carotenoids (Zhu and Jackson, 2015). Both DuPont (Sharpe et al., 2014) and Microbia (now part of DSM) (Bailey et al., 2012) have studied on using *Y. lipolytica* as the host for carotenoid production. In DuPont’s research, coexpression of codon-optimized *CrtE, CrtB, CrtI*, and *CrtY* in an omega-3 EPA-producing strain produced β-carotene to 5.7 mg/g in biomass, which contributed to 64% of the total carotenoids. The feasibility of engineering *Y. lipolytica* to produce canthaxanthin and astaxanthin has also been demonstrated (Sharpe et al., 2014). Microbia also engineered *Yarrowia* to produce β-carotene and demonstrated that the β-carotene produced by *Y. lipolytica* is the same quality as other commercial products on the market (Bailey et al., 2012). Increasing lipid content by eliminating β-oxidation is helpful to improve carotenoid production. For example, overexpression of codon-optimized *CrtE, CrtB, CrtI*, and *HMG* in a mutant (*pox1*–*pox6* and *gut2*) improved the strain’s lycopene production to 16 mg/g DCW in fed-batch fermentation (Matthaus et al., 2014). Further optimization of gene copy number, promoter strength, and fermentation conditions successfully led to 4 g/L β-carotene production, up to 50 mg/g DCW (Gao et al., 2017).

## Conclusion

Microbial oil-based biodiesel is one type of biofuels that demonstrated great potentials to replace fossil fuel in future. *Y. lipolytica* is a non-conventional, oleaginous yeast that is able to produce high levels of intracellular lipids under fermentation conditions, thus is considered as a potential host strain for economically viable biodiesel production. There are many economical plants as renewable resources that are rich in lignocellulose, inulin, molasses, and/or starch to provide C5 and C6 sugar monomers for further bioconversion into microbial lipids. Metabolic engineering of *Y. lipolytica* has initially focused on direct utilization and conversion these complicated carbon sources to fatty acids. Further engineering in the FAS pathway requires efficient gene expression in each key step and the cofactor balances, especially for NADPH generation through the PPP pathway. Minimizing or eliminating the fatty acid oxidation activities, especially β-oxidation, is critical to maintain a high-lipid yield and avoid the loss of the produced lipids. Direct production of biodiesel, either in FAEE or in FAME format, is still a challenge to make the process cost effective though significant progress has been made. Secretion of the produced FFAs can be an important direction to simplify both fermentation and downstream recovery processes, thus to further lower the overall operating cost. Novel fermentation technologies for high-throughput strain screening in micro- or mini-scale bioreactors, medium and process optimization, and scale-up should be developed to establish a biodiesel manufacturing platform. A two-stage continuous fermentation process that combines continuous cell growth and continuous lipid production has demonstrated promising economics for future biodiesel or lipid-related production. To increase the economic flexibility, development of high-value products derived from or related to lipids, such as omega-3 fatty acids and carotenoids, should be integrated into the microbial oil-based biodiesel technology platform.

## Author Contributions

The author confirms being the sole contributor of this work and approved it for publication.

## Conflict of Interest Statement

The author declares that the research was conducted in the absence of any commercial or financial relationships that could be construed as a potential conflict of interest.
